# Autism, autonomy, and authenticity

**DOI:** 10.1007/s11019-019-09909-3

**Published:** 2019-06-04

**Authors:** Elisabeth M. A. Späth, Karin R. Jongsma

**Affiliations:** 1 Department of Ethics and History of Medicine, University Medical Center Göttingen, Humboldtallee 36, 37073 Göttingen, Germany; 2 Department of Medical Humanities, Julius Center, University Medical Center Utrecht, Utrecht University, PO Box 85500, 3508 GA Utrecht, Netherlands

**Keywords:** Autism, Autonomy, Negative and positive liberty, Justice, Authenticity, Adaptive preferences

## Abstract

Autonomy of people on the autism-spectrum has only been very rarely conceptually explored. Autism spectrum is commonly considered a hetereogenous disorder, and typically described as a behaviorally-defined neurodevelopmental disorder associated with the presence of social-communication deficits and restricted and repetitive behaviors. Autism research mainly focuses on the behavior of autistic people and ways to teach them skills that are in line with social norms. Interventions such as therapies are being justified with the assumption that autists lack the capacity to be self-reflective and to be “author of their lives”. We question this assumption, as some empirical research shows that autists are aware of their strengths and are critical about social norms, we take this as a starting point to reconsider the beliefs about autistic people’s capacities. As a theoretical framework, we draw on Berlin’s idea of positive and negative liberty as he clearly distinguishes between one’s own developed preferences and the simple absence of interference. By drawing on the concept of positive liberty, we illustrate that a lot of autists are aware of their own needs, and usually do not deny their own needs, values and interests. This makes them less prone than non-autistic people to adapt their preferences to external influences, which might be seen as sticking to an authentic way of living. Our analysis shows that many autists are hindered to be(come) autonomous due to unjustified interference, unreflected assumptions about their self-determination, or by paternalistic actions. These observations contribute to a better understanding when help and interference are justified and a more differentiated understanding of autonomy of autistic people.

## Introduction

Autism is commonly understood as a spectrum disorder, which refers to a heterogeneous group of behaviorally-defined neurodevelopmental disorders associated with the presence of social-communication deficits and restricted and repetitive behaviors. While the prevalence of autism seems to be rising (Hollin 2017, p. 210) and diverse sciences direct to different explanations concerning the causes of autism (Autism Research Foundation 2018), the way we should understand autism, is still not well understood and remains understudied. Autonomy is an essential concept in medical ethics that deserves special scrutiny in the understanding of conditions such as autism, as it constitutes a prerequisite to make one’s own decisions and to create the life one wants to lead (Jennings 2016).^1^ Judgements about being allowed to shape one’s own life, or whether one needs to be protected, need to be made very carefully, and require a clear conceptual analysis. The main approach to study autism has primarily been focussing on the behavior of autistic people (cf. Bumiller 2008), including their ability to empathize with others and to adapt themselves to societal conventions. Furthermore, in cases of autism, autonomy is often conflated with other concepts (such as “best interests”) without an adequate reflection upon its implications for amongst others decision-making in the medical context and guardianship (Graber 2017). A diagnosis of autism seems to be a reason to be skeptical about the autonomy of the autistic person (Parsi and Elster 2015, p. 3). At the same time, autistic people are often described to be talented, singleminded and original, which is associated with the concept of authenticity; autistic people are not easily influenced or steered by others (Klauß 2005, p. 3). These perspectives pose an interesting starting point for our understanding of autism as they seem to direct in different directions: while autistic people may be constrained in performing daily life tasks and do not simply obey social conventions, they are otherwise individual and independent in their own way by following their genuine interests.

The aim of this paper is to raise awareness and steer up the debate on the conceptual understanding of autonomy in the context of autism. In order to provide a such a conceptual starting point, we follow Berlin’s distinction between positive and negative liberty (1969). Berlin distinguishes between one’s own developed wishes or preferences (positive liberty) and the (non-) interference by others (negative liberty). Both kinds of liberty constitute relevant conditions for autonomy, this allows us to analyze fostering and hampering factors for autists’ autonomy. Moreover, this enables a normative analysis that departs from the narrow behavior-oriented approaches to understand autism. By drawing on several empirical studies, we include the perspectives and experiences of autistic people in order to provide an empirically-informed notion autonomy in this context. In the following we will discuss: (1) the concepts positive and negative liberty and what the denial of the respective liberty entails, (2) to what extent autistic people are denied negative liberty and positive liberty, (3) the ethical implications of how we think about autistic people’s autonomy, addressing their values and actions by taking a critical stance towards Berlin’s approach.

## Conceptual framework: two concepts of liberty

Following the Kantian tradition, Berlin defines autonomy in relation to the capacity to rational thinking or decision-making: the autonomous person wishes to “be a subject, not an object” and is self-aware. This implies that one is aware of one’s strengths and shortcomings and takes responsibility for one’s choices (Berlin 1969). The conditions necessary for autonomy are a complex interplay of necessary values: consciousness, acceptance, understanding and knowledge about oneself and the world. The differentiation between negative and positive liberty serves as a framework concerning epistemological and ontological ideas on the conditions under which autonomy is exercized. While the concept of negative liberty places the focus on the absence of obstacles for a person to grow, the concept of positive liberty concentrates on the presence of internal processes of “organizing” one’s own values, and goals (cf. Takala 2007). The notion of negative liberty implies that one is free from any interference by others (Berlin 1969, p. 371). This can be illustrated by an “open-door” metaphor; any door should be open, irrespective of which door one chooses in the end. Negative liberty can thus be understood as a “precondition for self-realization” (Nys 2004, p. 217), as it includes the idea that opportunities are given to us. Positive liberty, in contrast, refers to the idea to be free to be or do something. Importantly, positive liberty “derives from the wish on the part of the individual to be his own master” (Berlin 1969, p. 131), which falls back onto the willpower of the person to organize one’s life according to one’s own goals and interests. Self-determination is used by Berlin as a compatible concept to circumscribe positive liberty, as it mirrors the extent to which one actually takes advantage of the possibilities to pursue what one plans or desires. In sum, positive liberty can be regarded as the inner process of weighing values and plans, while negative liberty is a reflection of external conditions. Autonomy might be hindered, or denied, in terms of realizing what one actually pursues, which implies a normative bias. On the one hand, negative liberty is denied when others interfere in someone’s (life) decisions, so that the person would not be self-determined, but determined by others. Based on one’s interpretation of the autist’s (in)dependence or (in)competence, another person may feel entitled to decide for the person. Negative liberty is denied when options are restricted and one is directed towards choosing the option others think is good for that particular person. From an ethical perspective, this leads to the question on which grounds others decide when to interfere and whether these grounds are justified. Positive liberty, in contrast, is expressed as self-denial, while being co-shaped by societal structures and influences of other people; Berlin himself points to the danger of authoritarian regimes, in which one is actually not free in the positive liberty sense because one is “deceived” by the guarantee of non-interference (negative liberty), but one can still be constrained in personal development. Essentially, there can be a misfit between the available and the required resources to achieve one’s true goals and interests (cf. adaptive preferences, a concept we will clarify under the section positive liberty). Drawing on these theoretical concepts, we will analyze in the following how autonomy of autistic people could be understood and what this implies in respect to how non-autists should understand them.

## Denial of negative liberty

Many autistic people need practical help with daily tasks related with self-organization, such as planning things ahead for activities such as traveling or shopping. Such help can be arranged for several domains, which is reflected in different forms of guardianship that may support everyday decisions (e.g., decisions for food, clothing etc.), or medical and financial decisions (cf. ibid., p. 961).

The observation that autistic people generally need more (practical) support in comparison to non-autists is sometimes taken as an a priori argument against their potential of be(com)ing autonomous (Bloom 2015). This is reflected in the conflation of autonomy and Theory of Mind (ToM). This ToM and similar observations (e.g., studies on weak central coherence theory, cf. Frith 2003) as well as critical reflections upon these studies (e.g., McGuire and Michalko 2011; Milton 2014; Verhoeff 2015), seem to indicate that autistic people do not grasp other people’s goals and intentions, nor that they are able to develop or organize a way of life according to their preferences, goals and interests (Lyons and Fitzgerald 2013, p. 752). From the observation that autistic children do not grasp others’ intentions and perspectives as non-autistic people do, it is assumed that they also do not grasp their own intentions and perspectives. This line of thinking fuels some parents, professionals and advocacy organizations to suppose that autistic people do not actually know what is “good” for them and that their behavior negatively affects others and therefore need to learn certain skills to cope with their environment. This becomes problematic, when interference (denying negative liberty) becomes the default without critical reflection whether that is justified or when the desirability of interference is assumed due to ill fitting with social norms/desirable behavior. For instance, when an autistic child openly objects towards taking medicine, parents and legal authorities often justify the continuation of treatment, based on the positively valued changed behavior of the child (e.g., being less aggressive) (see Benson and Pinnaro 2015).^2^ This is an interesting case to distinguish between helping and interfering; how we judge this case depends largely on how we understand autism and autonomy. If the child cannot decide for himself, because his preferences are irrational, he lacks the capacity to reflect or understand the consequences of his actions, the parents should help to choose the best option (whether medicine to be less aggressive is always the best option and is always helping, is another questions to be considered). If we understand the child as having a growing capacity to become autonomous, the parents may need to help the child understand his own preferences, his own beliefs and help reflect upon what would be good for him. Trying to understand the preferences of the child, by taking these serious and into consideration in decision making helps him to become autonomous and simultaneously may help to find more proportionate, less radical and better suiting (treatment) options. If the child is (becoming) autonomous, ignoring his growing capacities, preferences and simply providing the medicine would be interference. The default in the context of autism seems to be that this distinction—between interference and help—is not made. In a situation with socially undesirable behavior, the default is that others decide. This is problematic, not only because it hampers the child to become (more) autonomous; paternalistic actions like these also hamper the understanding why the child behaves in a certain manner and what may truly help him. Another example that indicates that there is a strong “behavior standard” for deciding what is good for autists can be found in applied behavioral analysis (ABA), a contested therapy that is aimed at teaching autists to display social behavior, basically focuses on “turning” autists into “social thinkers” (Richman 2015, p. 356). ABA entails that autists are trained to adapt to social conventions, for example learning to shake hands (ibid.). Some empirical studies also describe other examples such as the use of egg-timers to teach autistic people to eat slowly so that others do not feel disturbed and become willing to integrate the autistic person in the group (Hendriks 1998). In a school context, Sjödin (2015) describes an autistic student, whose interests and values are systematically disencouraged by school authorities: although she shows her strengths in taking initiative to structure and explain specific topics, teachers point out her weaknesses with regard to her style of working, such as her inability to follow the timetable and rules in class (Sjödin 2015, p. 91).

Non***-***autists often intervene in autists’s decisions so that they cannot be called to be “free from interference”. For autists it is assumed that they (1) do not know what is good for them (2) need to adapt to rules and social conventions and become “equal” members of society (3) have limited capacities and resources to develop themselves. While many autists need help with daily tasks or within certain social situations, interference with their lives is not automatically legitimized; help offered by non-autists may be useful to overcome barriers, as opposed to interference that may hamper the autist to pursue his own goals and thereby harm the original idea of negative liberty. As pointed out by Robeyns, constraining options or attempts influencing decisions are “likely to happen much more with autistic persons” (Robeyns 2016, p. 7), because autists might have issues to explain themselves in the immediate situation. In the following, we will argue that there are instances where autists show that they are free (in the positive liberty sense), which also has relevance for respecting their negative liberty.

## Denial of positive liberty

*Berlin’s concept* of positive liberty—being free to do something—describes the possibility to join in activities that are in line with values we actively and consciously proclaim. Autonomy, as an act of deciding for or against certain practices when one does (not) embrace the values or normative premises embedded in those, is an expression of the state of positive liberty and an authentic life. To deny one’s positive liberty means that one cannot live a life that is based one’s own goals and values. Instead, one uncritically adopts certain given opportunities (societal-structural level) and social cues (normative level). In the context of oppressive conditions, this may pose a threat to authenticity, and consequently, autonomy and well-being of a person, which is reflected by the concept of as adaptive preferences.^3^

First, adaptive preferences reflect a process in which one convinces oneselve to adapt one’s wishes and values to the circumstances under which one lives, thereby disregarding own goals and values. To cope with a situation in which one is not given much choice, one may downplay or even deny that one is not well in the given situation. The respective person then lacks autonomy in the sense of not being able to live their life in an authentic, self-determined way and consequently may seriously harm their own wellbeing. In the context of autism, we question whether the split into authentic and non-authentic preferences is applicable “on the same scale” as to autistic people. Autists generally have a set of key interests or habits as part of their personality/identity from which their everyday as well as “higher goals” preferences naturally follow. Some have assumed that autistic people are not self-aware and do not understand certain social pratices (e.g., Livingston and Happé 2017), but there is evidence that autistic people are able to prioritize their own values and are able to make choices without being bothered by social commodities and peer-pressure. Brownlow (2010) describes for example, how autistic people “mock” non-autistic behavior patterns, like placing much focus on social standing by comparing oneself with. According to Shelly (2004), autistic people, for example, do not identify themselves with their gender from early age onwards, as a category of making sense of their character and “place in society” (Shelly 2004, p. 7), in contrast to many non-autistic children. Autistic people do not simply accept categories and conventions, for the sake of being socially accepted. Simultaneously, it is very important to autistic people to have a meaningful task (cf. Brownlow 2010; Sjödin 2015) that relates to them as individuals; some argue that contributing to society with their abilities and personality is a “key to happiness” for them (Post et al. 2017, p. 96). These examples illustrate that autists are able to and even prefer to give their life a certain direction and spend their time with goals and values that are in line with what they value. Autists seem to be less prone to be influenced by habits or options others embrace, which makes them authentic and better “guardians of their own interests” than non-autists.

The second problem related with the issue of adaptive preferences concerns the assessment of autists’ autonomy and whether the lifestyle is evaluated as authentic: while non-autists often change their preferences rather unconsciously to their environment and meet social expectations (adaptation), autists rather actively choose options with which they distance themselves from others in the end (compensation). This insight is strengthened by observations of Davidson and Henderson who propose that autistic people develop and consciously apply a “qualified deception repertoire” (Davidson and Henderson 2010, p. 161). Crucially, autistic people are aware that they cannot or do not want to fulfill a demand and therefore deliberately find a way out to prevent that others regard them as impolite or dismissive. While it may look from a non-autistic perspective that autists, for instance, “overcome” themselves, autists do not disregard their own needs and wishes, but rather take a critical step in order to judge non-autistic people’s insistence on changing the autist’s preference.^4^ This can be best understood as *compensatory*, not adaptive behavior. The fact that autists tend to regularly apply compensatory strategies is recognized, as many autists develop stress, anxiety and depression by pretending (Livingston and Happé 2017, p. 15) to be social or to be concerned about things they normally do not care that much about. Even if living a “double life” is stressful, and may lead to similar consequences as adaptive preferences, one may wonder whether we should assess it the same. The crucial aspect of adaptive preferences is that one disregards one’s own preferences and wishes, and forces oneself to adapt to societal norms. For autists it seems a different matter, they can choose to adopt their own preferences or choose to follow societal norms, but they stay reflective on the desirability to adapt such norms, as they recognize that living a non-autistic life does not make them happier (Post et al. 2017).

## From liberty to autonomy

The insights attained by applying the concepts of negative and positive liberty help to understand how autists’s autonomy is currently approached and how to better conceptualize it. On the one hand, the concept of autonomy seems to be twisted: instead of understanding autonomy as setting and developing own rules and values, autonomy is measured and supported in terms of *adapting to* a prevailing system or fulfilling certain *standards*. On the other hand—illustrated by the concept of adaptive preferences—leading an autonomous life in the sense of authenticity, seems fulfilled for many autists, for whom their level of autonomy is dependent on *their own* idea of what is good for them and how they can integrate themselves best in society. Non-autists seem to fall easier for the trap of adaptive preferences, because of their social sensitivity, they may adopt social values without being self-reflective or by adopting authentic values. Autists do not simply adopt values others “preach” because they stick more strongly to their own values and preferences, partly because certain conventions “are irrelevant to them” (Bumiller 2008, p. 977). Autists are very much aware of the fact that adapting to options others assume to be suitable for autists too, in fact do not enhance their-well-being (cf. Spillers et al. 2014, p. 257). However, this seems to be underestimated by non-autists, establishing therapies like ABA or deducing claims on autist’s autonomy from theories like ToM; one can wonder what actually is achieved with social obedience therapy and certain interferences in general, because *displaying* social behavior is not the same as *becoming* a social person: wanting and deciding to be a social person. These observations have normative implications as intervening with autists’ behavior in such manners can hamper autists to be(come) autonomous: acts of non-autists’ with “good intentions”, eventually do not alter the well-being of autistic people (cf. Robeyns 2016; Rodogono et al. 2016) but exhaust their personal resources to pursue what matters to them.

## Discussion

As mentioned earlier, autism has been described mainly from a behavioral perspective, from which slippery-slope-like conclusions are drawn: from the observation that autists are dependent on others for some tasks, it is assumed that they also cannot choose for themselves and others are in a better position to judge what is good for them. In practice, non-autists eventually assume autists are being constrained to live autonomous lives. We are well aware that we cannot draw any general conclusions about the autonomy of such a heterogenous group, but we argue here that if we want to assess the autonomy of autistic individuals, we need to reconsider the yardstick we use for it. Our theoretical approach towards understanding autists’ autonomy by their authenticity is not meant to trivialize the ongoing care some autists need and are dependent on. We rather aim to raise awareness concerning the interpretation of such dependency and its ethical implications. In the following, we first elaborate on the relation between negative and positive liberty, to illustrate the interdependence of the two concepts. Second, we discuss the implications of the heterogeneous collective of autists for understanding autists’ autonomy and we will indicate how the assessment of autonomy may be further developed.

### “Being free from” and “being free to” as interdependent concepts

Berlin was mainly worried that positive liberty is in particular prone to be “abused” by authoritarianism, due to the problematic possibility of (uncritically) adapting oneself to living conditions one is more or less forced to live with (see inner citadel, Carter 2018), or because minority views on the good life may be suppressed. Therefore, he suggested negative liberty to be a “better protection” for the individual to lead an autonomous life.

Autism can be regarded a counter-example for the assumption that negative liberty is a “better protection” than positive liberty. The concepts positive and negative liberty have in common that they represent an ideal of liberty as it is in the end only a representation of something we assume to be worthy of support. The difference is that in contrast to positive liberty, negative liberty ultimately is not defined by the relevant individual or groups that share a common interest, but by others representing a different opinion, role or standpoint (e.g., as an institution). The reasoning behind deciding for or against constraints is not straightforward and is, just as for positive liberty, influenced by normative premises or assumptions directly (negatively) affecting the well-being of the relevant person or groups.

Berlin’s concepts help here to understand two dimensions of autonomy, namely the ability to define for oneself what is important to one’s own life and also the lack of (societal) interference that could reduce or increase one’s options, but the concept of negative liberty leaves open in which context or situation someone *should* be free from interference. Nys describes negative liberty as a “too loose concept”, because not the number of opportunities is decisive but the quality or value of one’s opportunities (cf. Nys 2004). As the context of autism indicates, this truly makes the difference to really “support” autists in becoming autonomous, as opposed to interfering or non-interfering with their interests arising from their “self-rule”: if there is no acceptance towards values, goals and interests other than of those people setting the rules or guidelines (here: non-autists), alternative options are not provided and thereby a particular group or individuals (here: autists) are indirectly persuaded or forced to go “through the doors that are already there“. The difficulty of defining negative liberty for autistic people is also due to the observation that they sometimes do not understand non-autists’ “value system”; or they do understand it, but reject it. At the same time, to be autonomous and act autonomously, the value of opportunities is of particular significance for autistic people because their interests and therefore needs are particular, which have consequences for arranging and adapting contexts for autists (e.g., workplace). From an ethical standpoint, this means that the concepts of negative and positive liberty need to be understood in *mutual acknowledgement*: autonomy is facilitated by those who are informed by the other(s) about their individual needs, while being at the same time dependent on the information the relevant individuals or groups share with them. As negative liberty per se cannot truly better protect freedom because it is not defined by the people who benefit from it. Negative liberty can only gain its legitimacy or authority in practice on the basis of positive liberty.

### Becoming autonomous: individuals and collectives

The negotiation which values are worth pursuing may be *expressed individually* but are *negotiated collectively* and autism basically revives the idea that individual freedom can only be reached via a collective. On an individual level, autists can be considered to be authentic as they do not hide what they like or dislike, but suffer from pretending to value things that non-autists appreciate. Given the heterogeneity of the group, of which some are judged to have an intellectual impairment (Matson and Shoemaker 2009; Goldin et al. 2014), the relevance of positive liberty rises beyond the individual level. As a collective, autistic people have successfully created a space for self-advocacy (e.g., Jongsma et al. 2017; DeVidi 2012), that allows them to negotiate their specific needs and interests, but also stand up for rights referring to any individual on the autism spectrum (e.g., http://www.ipsnews.net/2017/04/people-with-autism-have-right-to-autonomy-too/). Taking autists’ ability to be self-determined and to create collective action seriously, non-autists should question their prejudices of autistic people’s inability concerning their “self-rule”. On a conceptual level, this means that “true liberty” is not only to be gained via protective mechanisms (freedom from) but can and should be gained by the relevant individuals or groups by “going on the offensive” (freedom to) to gain support in realizing their needs and wishes.

### Limitations and outlook

Our analysis of negative and positive liberty has indicated important theoretical distinctions that point beyond behavioral approaches to understanding autism and has indicated the necessity to conceptually explore autonomy in the context of autism. Our theoretical analysis should be seen as a starting point to also critically reflect the usefulness of these very categories when defining concrete forms of support. Additional empirical studies are required to evaluate the potential of Berlin’s conception of autonomy. For this, the ICD categories might be a useful approach to apply and reflect upon the autonomy of differently impaired autistic people. Apart from that, we suggest to study collectives of autists in comparison to other groups who struggled and are still struggling for their liberty, against prescribed roles, and stigma (cf. Khader 2011). Furthermore, we have drawn on empirical work conducted by others to illustrate our more conceptual approach. Empirical research with autistic people will be beneficial to better understand their internal perspective, values and experienced liberty. An interesting way forward would be to further analyse whether negative liberty can be rightfully denied (Jaarsma et al. 2012; Post et al. 2017, p. 105). We suggest that empirical studies would provide a useful starting point, as the perspective of autists themselves should be included in answering this question.

Concerning to the conceptual notion of authentic preferences, further promising approaches may be found Harry Frankfurt’s notion of autonomy as he distinguishes between first-order and second-order preferences (Frankfurt 1988). For autonomy of autistic people, one of the remaining questions pertains to what extent they are free to choose their own interests and how deliberately they choose to follow these interests, in order to consider the interests authentic, or in Frankfurts terms, higher order preferences.

## Conclusion

Up until now, autonomy in the context of autism has been studied in relation with behavioral theory and has been understudied on a more conceptual level. On a practical as well as theoretical level, evaluating to what extent autistic people are autonomous is challenging for two reasons. First, autists may face difficulties to express themselves verbally, limiting our epistemic understanding of this condition from an internal perspective. Secondly, autistic people may prioritize values differently than non-autistic people While the actual assessment of whether someone is autonomous remains an individual assessment, our analysis indicates that both, autists as a group as well as individuals, are hindered to be(come) autonomous: in terms of positive and negative liberty, autists are restricted, based on assumptions of non-autistic people. The claim of autists to live an authentic life has consequences for our understanding on their autonomy, namely to question where and what sort of support is necessary. Berlin worried that positive liberty to be manipulated on a motivational level, but we have pointed towards a problem of equal importance: the normative assumptions underlying negative liberty may similarly be manipulated when they allow for interference rather than help; unjustified interference with life (choices) leads to a denial of negative liberty, while the help that autistic people need, should be aimed at helping them to live an autonomous life, or to become (more) autonomous. With respect to autists, this is true in particular as they have genuine goals, interests and talents they cannot pursue when they are deemed to be impractical, useless etc. or even harming others and/or society pushes them into a different direction because a different standard is taken at face value. Berlin’s conception of autonomy is helpful to draw a fine distinction between interference and support and how that relates to authenticity. With this, we could indicate different layers of injustices done to autists when autonomy is denied to them. Our study has aimed to refine and redirect the debate on autists’ autonomy to a more conceptual level. In order to further refine and assess whether individuals are autonomous and should continue to make their own decisions, other concepts of autonomy may have to be explored.

## Footnotes

1 For the following, we will avoid referring to a specific category of that spectrum: on the one hand, discussions on the DSM-5 indicate (cf. Spillers et al. 2014) that it is highly debated how autism can be diagnosed and exactly defined and on the other hand, autonomy has not actually been analyzed in respect to autists. Yet, it represents a concept which should be *generally considered* and should not be denied a priori because of a (specific) diagnosis of autism.

2 Although medical decision-making for minors is complex and heavily debated, many legal and ethical guidelines regard autonomy of children as a growing capacity, therefore their consent, assent or objection has to be considered or respected in medical decision-making (Jongsma et al. 2015).

3 A typical example for adaptive preferences is the “oppressed housewife”; in the 1960s women may have stopped questioning their prevailing role as a housewife, which was reduced to household tasks, and subordinated herself to the given structures in which women did not have equal opportunities and the same rights as men. The important normatively relevant observation is that women continued to positively evaluate their role, because they would have otherwise been confronted with barriers and/or (financial) risks and societal rejection, but may have felt unhappy and experiences the situation as problematic. The crucial stage of adaptive preferences is thus when societal norms become internalized so that the person uncritically identifies with them.

4 There is evidence that disrespecting an autistic person’s wishes in (medical) decision-making, including guardianship and proxy consent, is also prevalent beyond adolescence (Graber 2017).
